# Electrophysiologic Management and Considerations for D-transposition of the Great Arteries After Atrial Baffle Repair

**DOI:** 10.19102/icrm.2021.120805

**Published:** 2021-08-15

**Authors:** Akash H. Patel, Diana Glovaci, David Donaldson, Anthony C. McCanta

**Affiliations:** ^1^Department of Medicine, University of California Irvine Medical Center, Orange, CA, USA; ^2^Department of Cardiology, University of California Irvine Medical Center, Riverside, CA, USA; ^3^Department of Clinical Electrophysiology, University of California Irvine Medical Center, Orange, CA, USA; ^4^Department of Clinical Electrophysiology, Children’s Hospital of Orange County, Orange, CA, USA

**Keywords:** Atrial fibrillation, congenital heart disease, D-transposition of the great arteries, intra-atrial reentrant tachycardia, sudden cardiac death

## Abstract

This unique case highlights the electrophysiologic management and risk assessment of sudden cardiac death in a 35-year-old woman with a history of D-transposition of the great arteries status post–Mustard atrial switch repair.

## Case presentation

A 35-year-old woman with a history of D-transposition of the great arteries (d-TGA) status post–Mustard atrial switch surgical repair at six months of age presented for an electrophysiologic (EP) evaluation of palpitations. The patient had had an uncomplicated first pregnancy, yet in the postpartum state was diagnosed with atrial flutter (AFL) and atrial fibrillation (AF) requiring electrical cardioversion. Sotalol was initiated, then stopped due to gynecological bleeding, and the patient was subsequently maintained without medications.

During her second pregnancy, the patient experienced recurrent palpitations and was diagnosed with AFL. She was again trialed on sotalol but did not tolerate the medication. Ultimately, she required direct current cardioversion during pregnancy, and the child was urgently delivered via cesarean section. Her adult congenital cardiology team planned for an EP study and ablation after her recovery, but the patient moved to California prior to the procedure. She presented as a new patient to an adult congenital EP clinic to establish care 12 months after her cardioversion and birth of her child with symptoms of palpitations. A five-day event monitor revealed sinus arrhythmia with sinus rates between 64 and 141 bpm, with rare multifocal ventricular ectopic beats [94 total premature ventricular contractions (PVCs)]; one supraventricular tachycardia (SVT) noted to be atrial tachycardia versus interpolated PVCs/nonsustained ventricular tachycardia (VT) of 10 beats at a maximum rate of 113 bpm; and sinus node dysfunction (SND) with three pauses, with the longest pause lasting 2.1 seconds.

**Figure 1:**
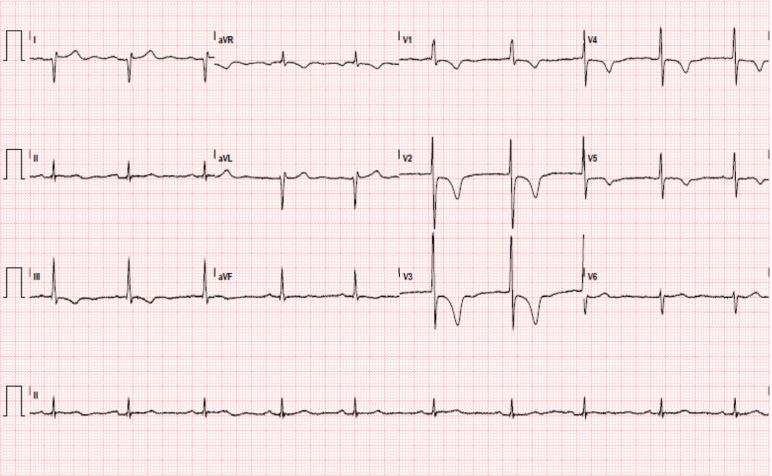
ECG showing sinus bradycardia with a heart rate of 58 bpm, a first-degree AV delay P–R interval of 207 ms, rightward axis, RV hypertrophy, and T-wave inversions in leads III and V1 to V5.

**Figure 2:**
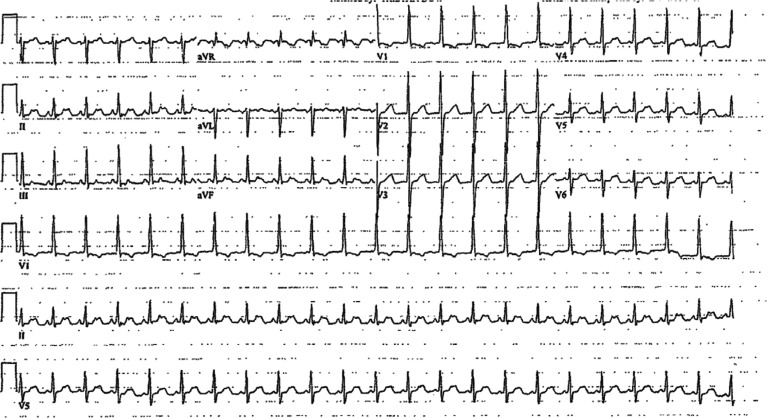
ECG showing IART/AFL with 2:1 AV conduction; ventricular heart rate of 133 bpm; and rightward axis, RV hypertrophy, and T-wave inversions in lead V1.

**Figure 3:**
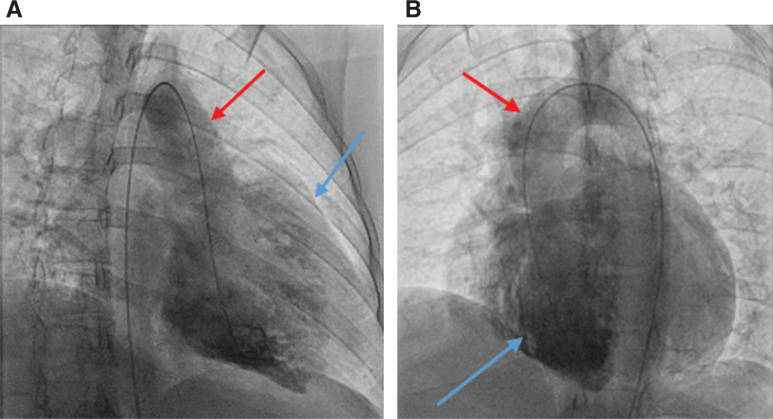
**A and B:** Still images taken from catheterization videos, showing the flow of contrast through the baffle structures. Right ventriculograms demonstrating mild RV dilation (blue arrow), no significant tricuspid regurgitation, and ventriculo-arterial discordance with the aorta arising anterior to rightward (red arrow) (d-TGA).

**Figure 4:**
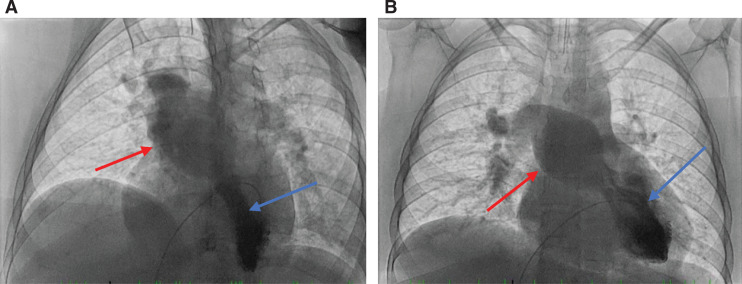
**A and B:** Still images taken from catheterization videos, showing the flow of contrast through the baffle structures. Left ventriculograms demonstrating ventriculo-arterial discordance with mild pulmonary valve stenosis and poststenotic dilation. The blue arrow shows contrast within the LV. The red arrow shows contrast entering the pulmonary arteries.

**Figure 5:**
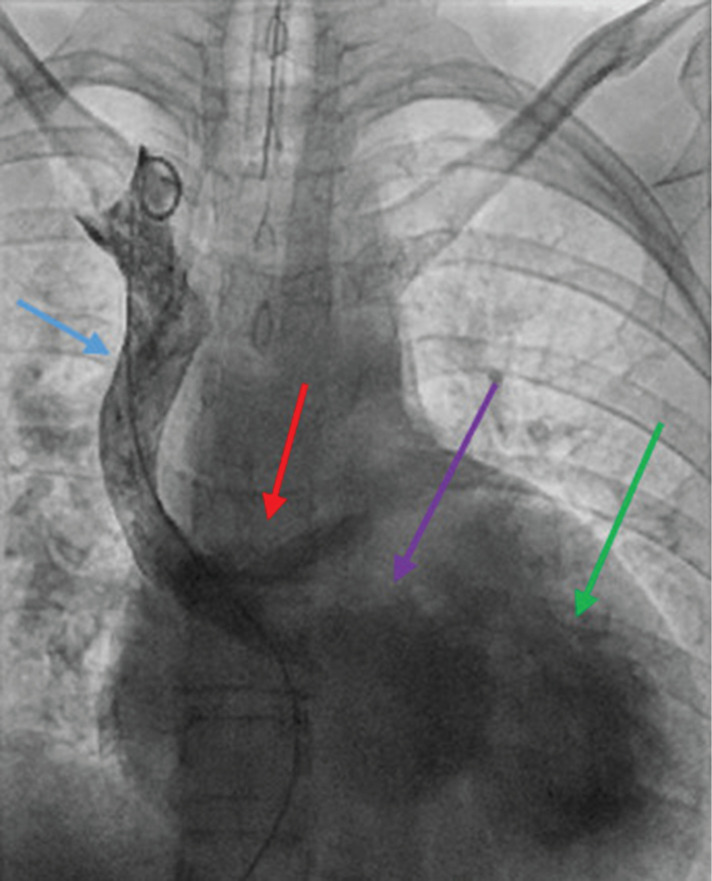
SVC (blue arrow) venogram demonstrating a patent superior Mustard baffle (red arrow), SVA (purple arrow), and LV (green arrow).

**Figure 6:**
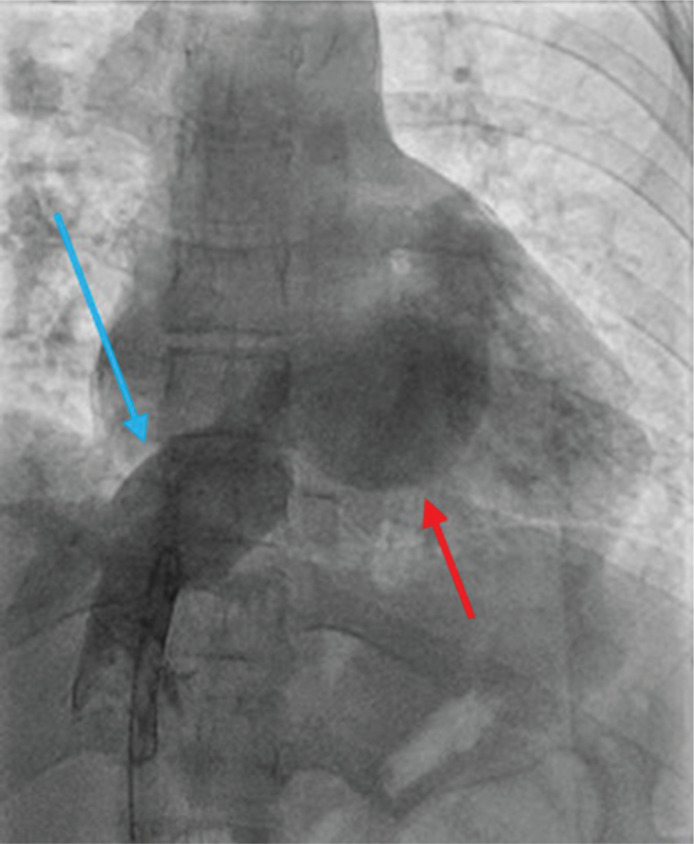
IVC venogram demonstrating a patent inferior Mustard baffle without baffle leak (blue arrow), SVA, and anatomic LA (red arrow).

**Figure 7:**
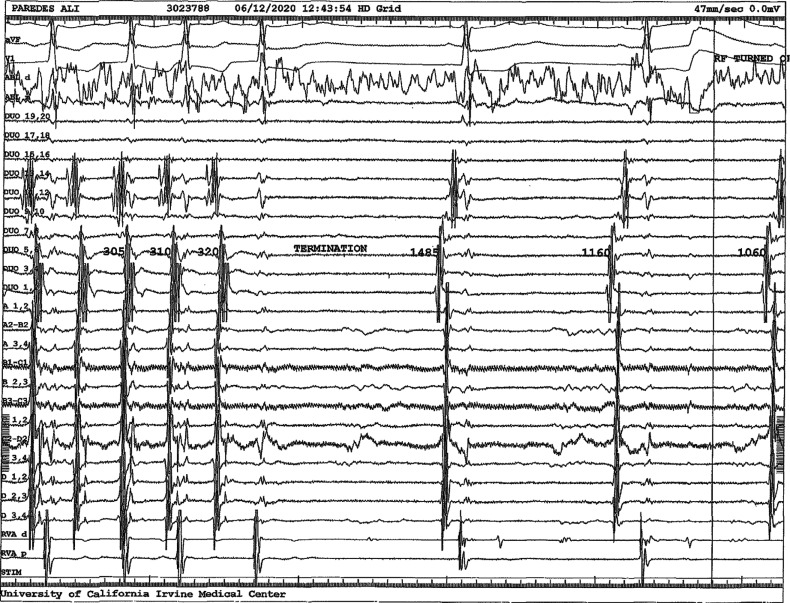
Intracardiac electrogram showing termination of the IART after radiofrequency energy was applied to the CTI from the PVA.

**Figure 8:**
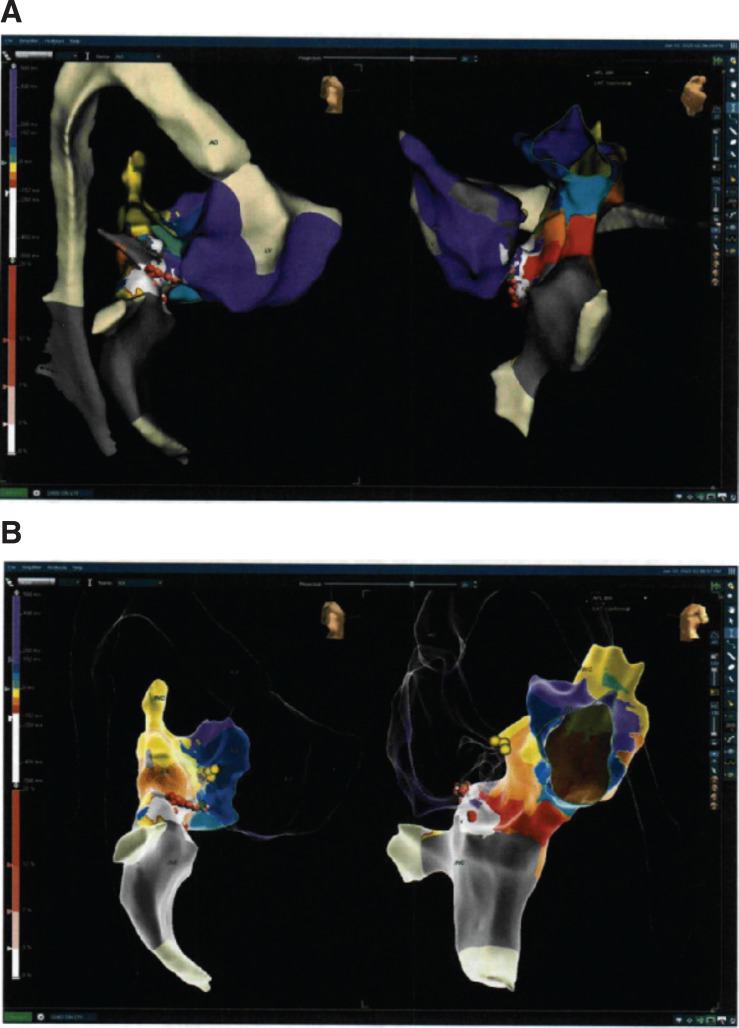
Three-dimensional electroanatomic map (ESI Velocity) demonstrating CTI-dependent IART with radiofrequency lesions (red dots) at the CTI and the specialized conduction system/His bundle in the usual anterior septal tricuspid location. Shown here are anteroposterior **(A)** and and lateral **(B)** views.

**Figure 9:**
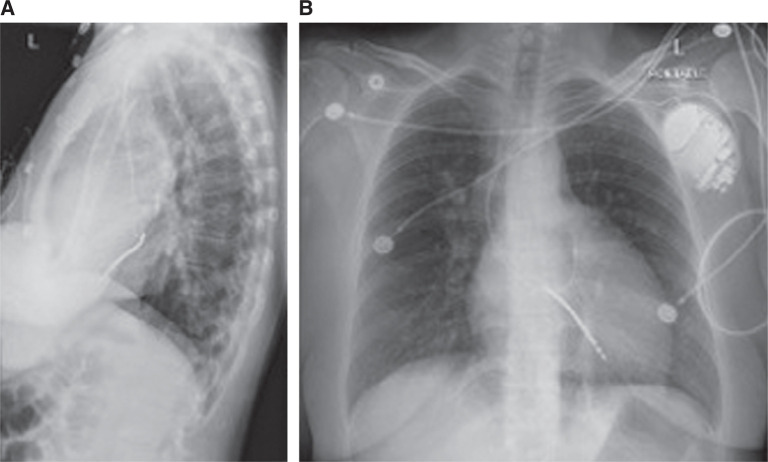
Frontal (right) and lateral (left) chest X-rays showing the single-coil ICD lead terminating in the LV and the atrial lead terminating in the native LAA in the SVA. The cardiomediastinal silhouette is stable and mildly enlarged. There was minimal atelectasis/scarring within the retrocardiac region and mild pulmonary vascular congestion. No obvious pleural effusion or pneumothorax and no acute osseous lesions were observed. Shown here are anteroposterior **(A)** and lateral **(B)** views, providing better visualization of the ICD leads.

The patient was referred to the University of California Irvine Medical Center for a comprehensive cardiac workup, including electrocardiograms **(Figures 1 and 2)**, right and left heart catheterizations (RHC/LHC) showing both hemodynamics and a beautiful overlay of the d-TGA anatomy **(Figures 3–6)**, an EP study **(Figures 7 and 8)**, and also consideration of placement of a dual-chamber implantable cardioverter-defibrillator (ICD) **(Figure 9)**. The differential diagnosis included SVT; sinus tachycardia; AFL; AF; atrioventricular (AV) nodal reentrant tachycardia (AVNRT); AV reentrant tachycardia; intra-atrial reentrant tachycardia (IART); paroxysmal atrial tachycardia, nonsustained VT; and frequent premature atrial, ventricular, or atrial/ventricular beats.

On the day of the procedure, the patient was hemodynamically stable, with an unremarkable physical examination with no jugular venous distension, her lungs were clear to auscultation bilaterally, had a normal heart rate and rhythm with a 2/6 murmur at the left midsternal border, and no pitting edema was noted.

The patient’s echocardiography results can be found in **Table 1**. RHC/LHC findings can be found in **Table 2**.

### Management (medical/interventions)

In this EP study, IART was induced with a cycle length of 300 ms with 2:1 AV conduction. Three-dimensional electroanatomic mapping was performed (ESI Velocity; Abbott, Chicago, IL, USA) utilizing a multipolar mapping catheter (HD Grid; Abbott) in the systemic venous atrium (SVA) and an irrigated-tip radiofrequency catheter (TactiCath; Abbott) positioned retrograde across the aortic valve into the RV and across the tricuspid valve into the pulmonary venous atrium (PVA). Entrainment was performed at the cavotricuspid isthmus (CTI) from the PVA and the SVA with a postpacing interval minus the tachycardia cycle length of less than 10 ms at each location, confirming the CTI-dependent IART. Radiofrequency energy applied to the CTI from the PVA resulted in the termination of the tachycardia **(Figure 7)**. There was a line of block across the CTI from the PVA to the tricuspid valve. The ablation catheter was then placed in the venous sheath into the SVA and radiofrequency energy was applied to the proximal CTI to the IVC. Bidirectional CTI block was confirmed with pacing lateral and medial to the radiofrequency line. No further tachycardia was inducible at baseline or on isoproterenol.

In addition to the EP study, a transvenous dual-chamber ICD with atrial antitachycardia pacing capability was implanted in the left pectoral area **(Figure 9)**. Interestingly, due to the atrial switch repair (Mustard) for d-TGA, the systemic venous anatomy diverts blood flow from the SVC to the native left atrium (LA) and subpulmonary LV. A 9-French (Fr) introducer was used, and a 9-Fr lead was threaded across the Mustard baffle into the LV apex. For the atrial lead, a 6-Fr lead was threaded through another 7-Fr sheath into the native LA appendage (LAA).

The patient was discharged home the following morning and was followed up with at 10 days, two months, and four months after her procedure. She remains asymptomatic with no atrial arrhythmias and no ventricular arrhythmias, with 11.2% atrial pacing and no appropriate or inappropriate shocks.

## Discussion

**Figure 10:**
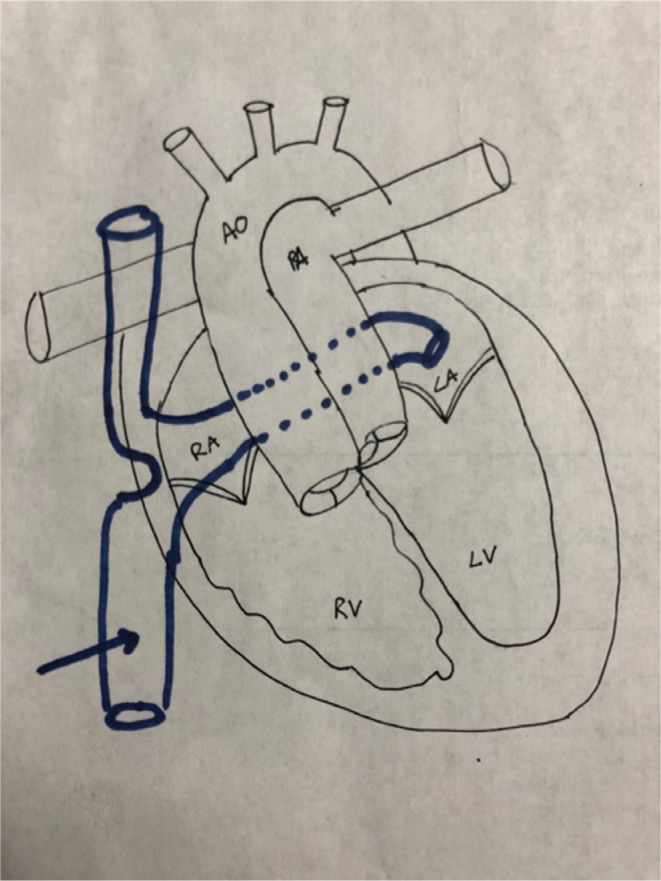
Anatomical sketch of the Mustard repair for surgical correction of d-TGA. Outlined in blue (blue arrow) is the baffle used to divert the caval blood to the left atrium (LA) to the left ventricle (LV) and subsequently to the pulmonary artery (PA). AO: aortic outflow; RA: right atrium; RV: right ventricle.

d-TGA is the primary condition among 5% to 7% of all congenital heart defects seen.^1^ The natural history of d-transposition shows that, without treatment, the mortality rate is 30% in the first week, 50% in the first month, 70% at six months, and 90% in the first year without repair. The first surgical “corrective” procedure was an atrial septectomy performed by Dr. Alfred Blalock; his assistant, Vivien Thomas; and his resident at the time, Dr. C. Rollins Hanlon, at Johns Hopkins University Medical Center to allow for an effective mixing of parallel circulatory systems.^2^ Several years later, the atrial switch procedures, Senning first in 1957, then Mustard in 1963, revolutionized the surgical management of d-TGA, thereby improving the 10-year survival rate to 90%.^2^ The Mustard procedure employs a baffle to divert the caval blood to the anatomic LA and LV. This venous blood is then pumped to the lungs through the pulmonary arteries **(Figure 10)**. This allows for the total correction of the d-TGA. Three years after the Mustard procedure was developed, Dr. William Rashkind developed a balloon septostomy, which was implemented in the first successful arterial switch in 1975 by Jatene et al. This increased the 10-year surgical survival rate to 97%.^3,4^

Notable postsurgical anatomical sequelae include baffle obstruction, systemic RV failure, and deconditioned subpulmonary LV. As surgical survival rates began to increase and more patients reached to adulthood, arrhythmias, primarily IART or AFL, became an increasingly well-recognized complication. It is thought that IART manifests from the multiple atrial suture line placed during surgical correction as well as reconstruction and scarring of the atrial tissue.^5^ Other atrial tachyarrhythmias seen in this population include AVNRT, focal atrial tachycardia, and AF.^5^

Due to the arrhythmogenic potential seen in these patients, many preprocedural considerations should be taken into account, including venous access (Rashkind atrial septostomy, historically up to 10 Fr in infants), AV node location, AV node access, coronary sinus access, CTI access, stable reference catheter, ventricular dysfunction, SVA, and PVA. Collins et al. demonstrated the location of ablation for IART in patients with congenital heart diseases, including after Mustard/Senning (n = 14 sites) and Fontan (n = 40 sites) procedures.^6^ Kanter et al. successfully conducted radiofrequency ablations of SVT substrates, including IART, in 11 patients with either Mustard (n = 9) or Senning (n = 2) operation.^7^ CTI-dependent flutter still remains the most common mechanism for IART in patients with atrial switch repairs for d-TGA. Importantly, because the baffle transects the CTI, ablation is required on both the systemic and pulmonary venous sides of the baffle in order to create a bidirectional conduction block across the entire CTI, as was required in our patient. One notable cause of death in these patients is premature sudden cardiac death (SCD). The incidence rate of this risk is 4.9 per 1,000 patient-years in SCD and 6.9 per 1,000 patient-years in non-SCD, with patients with d-TGA having a higher risk than those with tetralogy of Fallot in the Kaplan–Meier analysis for SCD-free survival.^8^ Interestingly, the presence of arrhythmic or heart failure symptoms in addition to documented AFL/AF was the highest predictor of SCD in patients with d-TGA [odds ratio (OR): 21.60 vs. 4.44, respectively], documented arrhythmia (OR: 3.47), SND (OR: 2.4), and AFL/AF (OR: 4.86).^9^ This patient had these risk factors for SCD and hence was offered a primary prevention ICD.

ICD placement in d-TGA patients is indicated for secondary prevention [hazard ratio (HR): 5.1], ventricular septal defect (HR: 4.3), moderate tricuspid regurgitation (HR: 4.1), corrected QT (HR: 1.02), and the inability to tolerate β-blockers (HR: 11.3).^10^ β-blockers have shown to offer a protective effect against primary ventricular arrhythmias among d-TGA patients. d-TGA patients who could tolerate β-blockers experienced fewer appropriate shocks compared to patients who could not.^10^ Preprocedural considerations for ICD implantation include baffle obstruction, baffle leaks, thin LV and risk of perforation, defibrillation vector, and atrial lead position in LAA extraction.

This unique case also highlights the EP management, arrhythmogenic potential, and risk of SCD in patients with prior atrial switch repair for d-TGA. It also highlights the comprehensive nature of treating IART in this subset of patients. This case demonstrates how multifaceted imaging and investigative modalities were used, including echocardiography, LHC, RHC, and EP studies, to grasp a better anatomical understanding needed to treat these arrhythmias while simultaneously assessing/reducing the risk of SCD.

### Follow-up

Our patient was seen postoperatively in the clinic and appeared to be doing well without recurrence of symptoms or significant arrhythmias noted on device interrogation and with excellent pacing and sensing parameters. The patient was seen again five weeks later without any complaints. ICD device counters were checked at each follow-up visit without any significant arrhythmia recurrences noted as of the most recent follow-up. She continues to be followed up with every two to three months on a regular basis for ICD interrogations and management.

## Conclusions

Atrial switch repair for d-TGA is an arrhythmogenic substrate. The risk of SCD is significant in d-TGA/atrial switch, and dysrhythmias are a risk factor for SCD. Successful EP management requires knowledge of the patient’s surgical/anatomical history and creativity.

## Figures and Tables

**Table 1:** Echocardiography Results

D-TGA with an intact ventricular septum
No evidence of superior vena cava (SVC) or inferior vena cava (IVC) stenosis (although SVC was not well visualized)
Moderate dilation and moderate hypertrophy of the right ventricle (RV)
Borderline diminished RV systolic function
Significant septal flattening from the RV into the left ventricle (LV) into diastole
Normal LV size and systolic function
Trivial mitral regurgitation
No evidence of significant pulmonary hypertension; magnetic resonance gradient estimated an LV pressure of 22 mmHg
Mild pulmonary valve stenosis with a peak gradient of 21 mmHg
Moderate pulmonary valve regurgitation
Mild tricuspid valve regurgitation
Left pulmonary artery (LPA) is moderately dilated
Right pulmonary artery (RPA) is not clearly seen

**Table 2:** Left and Right Heart Catheterization Findings

Mild pulmonic valve stenosis
Moderately dilated main pulmonary artery
RV and LV angiograms with normal wall motion and normal ejection fraction
No SVC or IVC baffle stenosis
IVC: 8 mmHg; SVC: 9 mmHg; baffle: 8 mmHg; LV: 42/9; right and left pulmonary capillary wedge pressure: 14 mmHg; LPA: 37/10 (20) mmHg; RPA: 32/10 (18) mmHg
RV: 123/16. No significant gradient between the RV, the ascending aorta, and the descending aorta
Saturations: IVC: 72%; SVC: 67%; LV sat: 67%; RPA: 64%; LPA: 69%; descending aorta: 97%; RV: 97%
